# Adenosine Triphosphate and Adenylate Energy Charge in Ready-to-Eat Food

**DOI:** 10.3390/metabo14080440

**Published:** 2024-08-07

**Authors:** Georgii Konoplev, Alar Sünter, Artur I. Kuznetsov, Piret Raudsepp, Tõnu Püssa, Lauri Toom, Linda Rusalepp, Dea Anton, Oksana V. Stepanova, Daniil Lyalin, Liubov Abramova, Andrey Kozin, Oksana S. Stepanova, Aleksandr Frorip, Mati Roasto

**Affiliations:** 1 Department of Photonics, Saint Petersburg Electrotechnical University “LETI”, 197022 Saint Petersburg, Russia; ovstepanova@stud.eltech.ru (O.V.S.); dolyalin@stud.eltech.ru (D.L.); osstepanova@etu.ru (O.S.S.); 2 Chair of Veterinary Biomedicine and Food Hygiene, Estonian University of Life Sciences, 51006 Tartu, Estonia; asynter@emu.ee (A.S.); piret.raudsepp@emu.ee (P.R.); tonu.pyssa@emu.ee (T.P.); dea.anton@emu.ee (D.A.); mati.roasto@emu.ee (M.R.); 3 AS Ldiamon, 50411 Tartu, Estonia; artur.kuznetsov@ldiamon.eu (A.I.K.); aleksandr@ldiamon.eu (A.F.); 4 Institute of Chemistry, University of Tartu, Ravila 14A, 50411 Tartu, Estonia; lauri.toom@ut.ee; 5 Research Institute of Fisheries and Oceanography, 105187 Moscow, Russia; abramova@vniro.ru (L.A.); kozin@vniro.ru (A.K.)

**Keywords:** nutrition nucleotides, adenylate energy charge, nuclear magnetic resonance, liquid chromatography, nucleotide salvage, meat, fish

## Abstract

It is commonly accepted that dietary nucleotides should be considered as essential nutrients originating mainly but not exclusively from meat and fish dishes. Most research in food science related to nutrition nucleotides is focused on raw products, while the effects of thermal processing of ready-to-eat food on nucleotide content are largely overlooked by the scientific community. The aim of this study is to investigate the impact of thermal processing and cold storage on the content of dietary nucleotides in freshly prepared and canned ready-to-eat meat and fish food. The concentrations of ATP, ADP, AMP, IMP, Ino, and Hx were determined using NMR, HPLC, FPMLC, and ATP bioluminescence analytical techniques; freshness indices *K* and *K*_1_ and adenylate energy charge (AEC) values were estimated to assess the freshness status and confirm a newly unveiled phenomenon of the reappearance of adenylate nucleotides. It was found that in freshly prepared at 65 °C ≤ T ≤ +100 °C and canned food, the concentration of free nucleotides was in the range of 0.001–0.01 µmol/mL and remained unchanged for a long time during cold storage; the correct distribution of mole fractions of adenylates corresponding to 0 < AEC < 0.5 was observed compared to 0.2 < AEC < 1.0 in the original raw samples, with either a high or low content of residual adenylates. It could be assumed that heating at nonenzymatic temperatures T > 65 °C can rupture cell membranes and release residual intracell nucleotides in quite a meaningful concentration. These findings may lead to a conceptual change in the views on food preparation processes, taking into account the phenomenon of the free adenylates renaissance and AEC bioenergetics.

## 1. Introduction

Among other important trends in food science and technology, there are two new conceptual proposals aimed at the development of new healthy and sustainable food products and processes that are worthy of special attention. One of them is related to the incorporation of free dietary nucleotides into the category of essential nutrients [1]. The second proposal is closely linked to the green concept and has been described as the “umamification of food facilitates the green transition” [2]. Umamification implies, among other innovations, the promotion of free nucleotides as essential nutrients. A new parameter “equivalent of umami concentration” (EUC) has even been introduced to characterize the synergistic effect between umami amino acids (mainly L-glutamate) and 5′-nucleotides on food palatability [3,4]. Thus, both proposals are inherently consistent and do not contradict each other. Such close attention to dietary nucleotides makes it necessary to provide deeper knowledge in this area of food science and nutrition [5].

Indeed, systematic scientific research on the unavoidable thermal effects on raw meat, fish, or vegetable materials during cooking is clearly insufficient. For example, in large critical reviews relating to food science and nutrition, no attention has been paid to nucleotides in hot processes [6,7,8]. Let us note the work in [9], which provides a thorough overview of the effects that occur during hot cooking, but only in terms of the occurrence of harmful aspects (thermal degradation) and does not consider neutral or potentially beneficial transformations. 

The purpose of this work is to somewhat fill the gap in terms of objective information on the effects of heating, with an emphasis on free nutritional nucleotides, especially adenosine-related compounds (adenylates). A constructive step in this direction would be a proposal to introduce indices of the conditions for thermally transformed fish and mammal meat in the form of ready-to-eat food, which can be freshly prepared, refrigerated, or canned. Such a nucleotide paradigm for processed food could be a part of the concept of essential nutrients [1]. The objects of this research—raw meat and fish—were processed for experiments in the same ways applied to normal food preparation and conservation processes to avoid extreme influences on them; for instance, homogenization was carried out only in rare cases for comparison purposes.

### 1.1. Nucleotide-Based Freshness Indices

To date, analytical methods for assessing the freshness and quality of raw fish and meat based on determining the concentration of nutritional nucleotides and nucleosides, i.e., adenosine triphosphate and its postmortem metabolites, have been very well developed and widely introduced into practical use. During the storage of fish, seafood, poultry, and mammal meat, irreversible transformations occur, as in the following chain:

ATP→ADP→AMP→IMP→Ino→Hx,(1)

where ATP, ADP, and AMP are adenosine tri-, di-, and monophosphates; IMP is inosine monophosphate; Ino is inosine; and Hx is hypoxanthine. Such transformations take place in muscle tissue due to reactions involving protein enzymes that are strictly specialized at each stage of the chain (1). These transformations occur intensively only at temperatures lower than ≈ +65 °C, which is the approximate denaturation temperature of all enzyme proteins. Our primary attention in this work is focused, however, on the effects occurring mainly in the temperature region of ˃ 65 °C. This is a strong specificity of this research.

In 1959, Japanese scientists proposed [10] the *K* index and Formula (2) as an indicator of the freshness and quality of frozen fish:

*K* = ([Ino] + [Hx])/([ATP] + [ADP] + [AMP] + [IMP] + [Ino] + [Hx]). (2)

This approach quickly disseminated throughout food science worldwide as a reliable method for the assessment of the freshness of not only fish but also poultry and mammal meat. Equation (2) was further simplified, taking into account the fast elimination of adenylates in fresh meat, and a new index *K*_I_ was introduced [11]:

*K*_I_ = ([Ino] + [Hx])/([IMP] + [Ino] + [Hx]). (3)

Since operating with the simplified Equation (3) requires half the amount of experimental data, the use of the *K*_I_ index has become even more widespread than the *K* index. The main argument for ignoring adenylates in Formula (2) is the fact that during the cold storage of meat or fish in a household refrigerator at temperatures T ≈ 0–4 °C, almost complete degradation of adenylates occurs within about 10 hours due to enzymatic processes. By this time, in vertebrate animals and fish, the most significant changes take place in the ratios of [ATP], [ADP], [AMP], and [IMP], so instead of ATP, ADP and AMP, IMP dominates with the concentration in the range of 5–10 µmol/g [10,11]. In this regard, the *K*_I_ index becomes a very convenient measure for determining the freshness of meat or many species of consumable fish in the most relevant storage time interval between 1 and 5–7 days.

Much greater accuracy (scrupulousness) is required during the analysis of absolute freshness, when all the metabolites in chain (1) and Formula (2) must be taken into account. Nowadays, this approach is increasingly relevant in real-life situations due to recent developments in the technical means of effective delivery, storage and rapid (shock) freezing of various meat, fish, and seafood products (see, e.g., [12]).

The case with absolute freshness is very important for human consumption of aquatic invertebrates: oysters [13,14,15], sea urchins [16], shrimp [17], other shellfish [18,19], and fish, which were frozen very quickly after catching [20]. In these objects, the adenylates pool can be preserved for a much longer time, e.g., in the work [14], frozen oysters were stored for 24 weeks and still retained sufficient amounts of adenylates. Naturally, in such a situation, it would be unacceptable to completely ignore these substances and therefore the analysis of freshness was carried out using not only the classic index *K* but also two new ones, i.e., *K*’ and AEC [13,14,15,16,18,20]. Specifically, a new drive had been undertaken by the authors of the pioneering work [13] and the index from a rather different science domain, namely, the adenylate energy charge (AEC), was successfully utilized for the assessment of freshness.

### 1.2. Adenylate Energy Charge

The adenylate energy charge (originally “energy charge” or EC) was proposed by Atkinson [21,22] as a fundamental metabolic control parameter:

AEC = ([ATP] + 0.5 × [ADP])/([ATP] + [ADP] + [AMP]).(4)

The need for the introduction of the index AEC was reasoned by the fact that the metabolic rates of chain (1) depend not only on the adenylate’s absolute concentrations but also on their molar ratios. Later, Atkinson and coauthors published the data on AEC in very diverse organisms and tissues [23]: in the list presented in the article, there are 91 items embracing bacteria, plants, seeds, few aquatic organisms, internal organs of animals, etc. The AEC approach or the adenylate energy system (Atkinson’s system) seems to be one of the most important laws in biological life. It is not surprising that AEC has come to be used as an index of seafood quality too. 

As far as we know, AEC was for the first time used straightforwardly as a quality parameter of food in the case of oysters stored for three weeks at 0 °C [13]. The authors concluded that AEC can be successfully employed as a freshness index for different oyster body parts. The basis for this conclusion was the slow decrease in the AEC value: in one of the experiments, the AEC decreased only by three times over three weeks [13].

One of the conclusions drawn in [23] was that “mammalian tissues are usually protected against extreme changes in external conditions, and they show uniformly high energy charge values, in the range between 0.75 and 0.9”. Despite the fact that very different biological objects were involved in the work [23], it reveals virtually no data on the AEC values in fish and animal muscles—the main objects of our research. Supplementary information about the AEC in widely consumable fishes, namely in the muscle tissue of almost alive trout (*Salvelinus fontinalis*) [24] and Atlantic salmon (*Salmo salar*) [25], was published later (both AEC ≈ 0.9). Outside the research works mentioned above, the information on AEC in fresh meat and fish is very scarce. The gap can be at least partially filled by the re-analysis of extensive experimental data on adenylate content in food products published in multiple original papers. This re-analysis, which is purely based on the literature data, was performed by the authors as a preliminary part of the presented research; the results are given in Section 3.1.

### 1.3. Nucleotides in Thermally Processed and Canned Fish

While the feasibility of using the nucleotide-based AEC, *K*, *K*’ and *K*_I_ indices as a reliable freshness indicator for raw fish, poultry and mammal meat is beyond question, much less attention has been given to the applicability of this approach to thermally processed freshly prepared ready-to-eat and canned food. Heat treatment has a noticeable impact on the relative distribution and dynamics of the dietary nucleotides in muscle tissue due to the effects of thermal deactivation of the enzymes involved in the ATP breakdown chain (1) and the direct thermal destruction and salvage of nucleotides. All these phenomena are extremely important and must be taken into account for a correct interpretation of nucleotide-based freshness indices in the case of ready-to-eat and canned food.

In the works [26,27], the minimum heating temperatures required for the full deactivation of enzymes were established for fish meat. For different species and different enzymes, the values were in the range of 60–75 °C. After thermal processing at higher temperatures, the concentrations of the ATP breakdown products remained stable during cold storage in contrast to raw fish. Similar results were obtained for other types of meat. This fact leads to a very important conclusion: nucleotide-based freshness indices have a slightly different meaning for thermally processed ready-to-eat food characterizing the freshness of the products used at the time of cooking rather than the freshness and storage period of a dish itself. This statement is valid for all types of meat and fish dishes except those prepared at relatively low temperatures: cold-smoked, dry-cured, salted, and sous vide products.

There are published cases that consider using the *K* and *K*_I_ indices to evaluate the freshness of fish raw materials from which canned food is produced and the freshness of ready-to-eat fish products undergoing heat treatment. Relatively few studies have been conducted on this topic, but the work carried out in this direction shows that it is quite possible to develop a technique that allows assessing the quality of raw materials used for canned and cooked fish based on the nucleotide content. The research presented in the article [28] has shown that the nucleotide content of raw and cooked fish may depend on whether it is farmed or wild and on its diet. Wild large yellow croaker had the lowest *K* and *K*_I_ values for raw and cooked fillets, but farmed fish had higher *K* values than wild fish when fed 44% fishmeal. The highest *K* and *K*_I_ index values were found when the diet contained 25% fishmeal.

Several studies have been aimed at a better understanding of the effect of heat treatment on the dietary nucleotides during high-temperature sterilization, which is an essential part of the canning process. The authors of the study [29] compared the changes in the *K* index and the concentrations of IMP, Ino and Hx in sashimi-grade Japanese mackerel and of acceptable freshness suitable for cooking before and after autoclaving and found that after autoclaving, the content of these nucleotides and nucleosides increases, as does the *K* index. Changes in the content of nucleotides after heat treatment were also noted in the other work [30]. It was reported that raw fish had the lowest *K* index, lowest Ino content and highest IMP content compared to heat-treated samples. When heated at 125 °C for 9 min, IMP content decreased, while Ino and Hx increased. The lowest IMP content and the highest Ino and Hx content were obtained when treated at 120 °C for 90 min. In addition to studying the effect of preservation processes on nucleotides, the authors have also attempted to determine the freshness of the raw materials from which three brands of canned sardines were made [31]. HPLC was used to determine the *K* index and equivalent biochemical age at 0 °C, 9 to 15 days.

It was reported earlier that an interesting effect opposite to the nucleotide thermal degradation, namely, nucleotide salvage was observed multiple times in fish, pork, and poultry meat after prolonged storage in a refrigerator or bought in a frozen state [32]. The biochemical nature of this effect is not completely clear yet; it manifested as a substantial increase in ATP, ADP, AMP, and IMP nucleotide content after thermal processing of fish and meat products, which were at the edge of spoilage. 

The effects of the thermal transformations of the dietary nucleotides and nucleosides (degradation or salvage) during heat treatment under conditions close to conventional domestic food processing or, even more importantly, high-temperature sterilization treatment before canning must be considered for a correct interpretation of the *K*, *K*_I_ and other nucleotide-based freshness indices for ready-to-eat and canned food. Neglecting these effects could be a potential source of misleading results, e.g., the *K* and *K*_I_ indices could achieve much higher values after heat treatment compared to raw products even for absolutely fresh meat and fish. 

## 2. Materials and Methods

Multiple samples of freshly caught or defrosted fish (26 samples of various species for one-time measurements and 66 raw and thermally processed samples from a single carp fish during the prolonged cold storage), canned fish (18 samples of various species) and mammal (2 pork samples) or poultry (6 chicken samples) meat were analyzed by the Fast Protein and Metabolite Liquid Chromatography (FPMLC), ATP bioluminescence, High-Performance Liquid Chromatography (HPLC), and nuclear magnetic resonance (NMR) spectroscopy to determine the basic nucleotides content, i.e., adenylates (ATP, ADP, and AMP), IMP, Ino and Hypoxanthine, and estimate the AEC and other freshness indices for raw meat and thermally processed ready-to-eat food. FPMLC and ATP bioluminescence were used mainly as fast and efficient analytical techniques for the preliminary analysis and selection of samples before employing more sophisticated and time-consuming methods such as HPLC and NMR. Most sample preparation and measurement procedures are thoroughly described in our previous publications [32,33]. Here, we only give some new or critical significance details. 

### 2.1. Fast Protein and Metabolites Liquid Chromatography (FPMLC)

**Sample preparation and heat treatment.** Both raw and thermally treated samples of fish, poultry, or mammal muscle tissue were analyzed by FPMLC. Heat treatment was performed by one of the following methods: relatively large samples (the pieces of muscle tissue) were hot steamed or heated in broths at different temperatures 65–120 °C in a conventional household pressure multicooker (Moulinex SERIE EPC51, Groupe SEB, Ecully, France); smaller samples were heated in broth in Eppendorf Tubes at different temperatures between 22 and 100 °C for 15 to 60 min in the Biosan TDB-120 dry block thermostat (Biosan, Riga, Latvia) or put in a microwave oven. Some samples were bought at a supermarket as ready-to-eat food, and heat treatment regimens applied to them by the producers remained unknown. The higher temperatures (T > 100 °C) in the pressure multicooker were used to imitate the sterilization process (autoclaving), which is essential for canning. Several trout samples were marinated with the acidic and complex (acidic + plant extract) marinades (pH = 2.24–3.03). These samples were used in NMR measurements in the Institute of Chemistry, University of Tartu (Tartu, Estonia), and no drastic difference from non-marinated trout has been observed. 

**Liquid extract preparation.** To prepare a liquid extract, 2 g of raw or thermally treated muscle tissue was manually cut into small pieces 1–2 mm in size and placed in a disposable test tube. About 6 mL of TRIS buffer (pH = 8.0) was added to the tube, and the content was mixed for 5 min using the MX-RL-E rotator from DLAB Scientific (Beijing, China) and centrifuged at 2300× *g* or 13,000 rpm for 10 min at room temperature (some samples were centrifuged at 4000 rpm). About 5 mL of the resulting liquid extract was taken out from the tube with a Luer-lock syringe and filtered with the Whatman^®^ GF/B glass fiber syringe filter (Product No. Z242195) from Merck KGaA (Darmstadt, Germany). 

**Chromatogram processing.** In total, 250 µL of the filtered extract was introduced into the PD-10 protein desalting column (Code No. 17-0851-01) from GE Healthcare^®^ Bio-Sciences AB (Uppsala, Sweden). FPMLC chromatograms were processed with an optoelectronic chemical sensor according to the protocol described in detail in [33].

### 2.2. pH Measurements

An intelligent pH meter of the YY-103 series from Shenzhen Yago Technology (Shenzhen, China) or the digital HandyLab680 pH meter (SI Analytics GmbH, Mainz, Germany) were used to measure the pH of raw fish meat or marinade solutions. The fish carcass was kept at room temperature for at least 20 min; then, an incision was made in the muscle tissue and the electrode of the YY-103 pH meter was inserted inside it. Steady-state readings were taken at room temperature. The pH meter calibration was regularly checked. 

### 2.3. ATP Biolomimscense

Rapid measurements of the ATP concentration were performed by the ATP bioluminescence assay using a luminometer of the EnSure series with the Aquasnap tests from Hygiena Int. (Watford, UK) or the Infinite 200 PRO microplate reader from Tecan Austria GmbH (Grödig, Austria) with the BioThema ATP Kit from BioThema (Handen, Sweden). The preparation technique of the liquid extracts was the same as for the FPMLC analysis; in the experiments with raw fish, an additional 1000-fold dilution of extracts (10 μL in 10 mL of TRIS buffer) was required due to an extremely high sensitivity of the technique. The ATP concentrations were read in relative light units (RLU). 

### 2.4. High-Performance Liquid Chromatography HPLC

Nucleotides and the related compounds were measured by a reverse-phase high-performance liquid chromatograph (HPLC) (Shimadzu, LC-20 Prominence, Kyoto, Japan) equipped with an SPD-20A (UV-VIS) detector. The Discovery@C18 HPLC Column (4.6 mm × 250 mm, 5 μm) was installed inside a thermostat compartment for use at a temperature of 30 °C. The mobile phase consisted of solvent A comprised 0.1 mol/L phosphate buffer (pH of 6.95) and solvent B acetonitrile. The program consisted of 100% A at time 0, 6.5 min 98% B, 10 min 95% B, 12 min 90% B, 14 min 75% B, 23 min 100% B. Flow rate of 1 mL/min. The samples were filtered through a 0.22 µm membrane and analysis was carried out by injecting 10 µL of sample and detection was monitored at 254 nm. Reference standards were used to calculate the quantities of ATP, ADP, AMP, IMP, HxR, and Hx.

### 2.5. Nuclear Magnetic Resonance (NMR) Spectroscopy

**NMR spectroscopy** was performed at two research centers: at the Institute of Chemistry of the University of Tartu (UT, Tartu, Estonia) and at the Laboratory of Biomolecular NMR-Spectroscopy, the Shemyakin and Ovchinnikov Institute of Bioorganic Chemistry, RAS (IBCh RAS, Moscow, Russia). 

In UT NMR measurements, were performed using the Bruker Avance III 700 NMR spectrometer equipped with a 5 mm Broadband Observe (BBO) probe. The ^1^H Larmor frequency was 700.08 MHz. The ^1^H NMR spectra were measured at 298 K with solvent suppression using the noesypr1d pulse sequence. The acquisition time was 3.67 s; the recycle delay was set to 4.00 s. NMR solutions were prepared by adding approximately 20% of deuterium oxide D2O (purity: 99.92 atom% D) containing 0.05% (*w*/*v*) sodium 2,2,3,3-tetradeuterio-3-trimethylsilylpropanoate TSP-d4 (acquired from Sigma-Aldrich, St. Louis, MI, USA, product number: 450510-25ML, batch number: MKBS0535V) to the aqueous samples. All spectra were referenced to TSP-d4 (0 ppm). Corrections to phase and baseline were performed with the Bruker Topspin 4.1.4 (Bruker, Rheinstetten, Germany). The free induction decays (FIDs) were multiplied by a line-broadening function of 0.1 Hz prior to Fourier transformation.

The NMR signals of AMP, ADP, ATP, IMP, hypoxanthine, and inosine were identified through spiking tests, which involved adding solutions of each compound and observing the corresponding increase in the NMR spectra.

Quantitative NMR (qNMR) was performed by adding a known amount of an internal reference (either adenosine 5′-monophosphate sodium salt (Sigma A1752-1G, batch number SLBN6801V) or inosine 5′-monophosphate (Sigma I2879-1G, batch number SLCC7039) to a measured aliquot of the meat broth. ^1^H qNMR spectra were obtained at a temperature of 298 K using solvent suppression with the noesypr1d pulse sequence. The acquisition time was the same as previously 3.67 s, but the recycle delay was set to 8.00 s. The concentrations of the determined ingredients in the broths were calculated using modified Equation (1) in [34]. 

At IBCh RAS ^1^H NMR spectra were acquired using the 600 Mhz Bruker Avance III NMR spectrometer (Bruker Biospin GmbH, Rheinstetten, Germany). The ^1^H NMR spectra were measured at 298 K with solvent suppression using the noesygppr1d pulse sequence Phasing, baseline correction and chemical shift calibration (using the TSP signal as reference, δ 0.0 ppm) of the frequency domain spectra was conducted using Bruker TopSpin v. 3.6.1 and spectral assignment was performed using the metabolite library provided by Chenomx NMR suite v. 10.0. The free induction decays (FIDs) were multiplied by a line-broadening function of 0.3 Hz prior to Fourier transformation.

**Sample preparation for NMR spectroscopy.** In UT in the NMR measurements, the same extracts as for the FPMLC chromatography were used. Between the FPMLC and NMR measurements, the liquids were stored at −18 °C for no longer than for a few days.

In the lab of the IBCh RAS, the water-soluble polar metabolites of fish samples were extracted with a 7.5% solution of trichloroacetic acid (TCA). For this purpose, 25 g of fish muscle was added to 50 mL of 7.5% TCA and homogenized using a vertical homogenizer. The homogenate was filtered through a paper filter. The filtrate was neutralized by a 9 M solution of KOH up to pH value of 7.4. The solution was filtered through the paper filter (Grade 1) and centrifuged at 4 °C and 12,000× *g* for 10 min. An aliquot of the resulting solution (500 μL) was transferred with a pipette to a standard NMR ampoule 5 mm in diameter and supplemented with 50 μL of a TSP (3-(trimethilsilyl) propionic–2,2,3,3-d_4_ acid sodium salt) solution in D_2_O at a concentration of 11 mmol/L (as an internal standard).

## 3. Results

### 3.1. A Comprehensive Analysis of the AEC as a Quality Factor in Absolutely Fresh Pork and Fish Based on Previously Published Experimental Data 

Experimental data on adenylate content in absolutely fresh pork were published in several original papers [35,36,37,38,39]. To categorize the pork quality, it was proposed in [35] to operate exclusively with [ATP] in raw meat and make a decision according to the following algorithm: “Normal”—[ATP] ˃ 1.5; “Pale, soft, exudative (PSE)”—[ATP] ˂ 1.0 and “Dark, firm, and dry (DFD)”—[ATP] ˂ 1.5 µmol/g [35]. For some reason, the determination of the pork quality according to the AEC index was not carried out in these research works. We have performed such an assessment based exclusively on the literature data for the first time using the AEC index for the pork samples with the categories “Normal” [35,36,37], “Red, Firm and Non-Exudative (RFN)” [36], “Acceptable” [37]), PSE [25,35], DFD [35], as well as for the freshest possible meat of pig breeds “Normal” and “Heterozygote” [38] and “Wild-type” and “Pigs harboring the AMPKγ3^R300Q^ mutation (AMPK)” [39]. The AEC values presented in Table 1 were estimated according to Equation (4) only for the pork samples stored no longer than 2 h after slaughter.

Table 1 clearly shows the following pattern: for the pork samples belonging to the high categories such as Normal and AMPK, the AEC values (0.85–0.93) are noticeably higher than for the categories “Low quality” (0.715), PSE (0.65) and DFD (0.6). This fact allows one to use the index AEC in practice for meat quality evaluation. In addition, it convincingly demonstrates the fundamental possibility of dealing with fresh pork in the adenylates paradigm. One can also notice that pork and other types of meat that have undergone shock freezing can remain very fresh for months. This realization greatly expands the number of food products that can be safely considered to be of the highest freshness.

**Figure 1:**
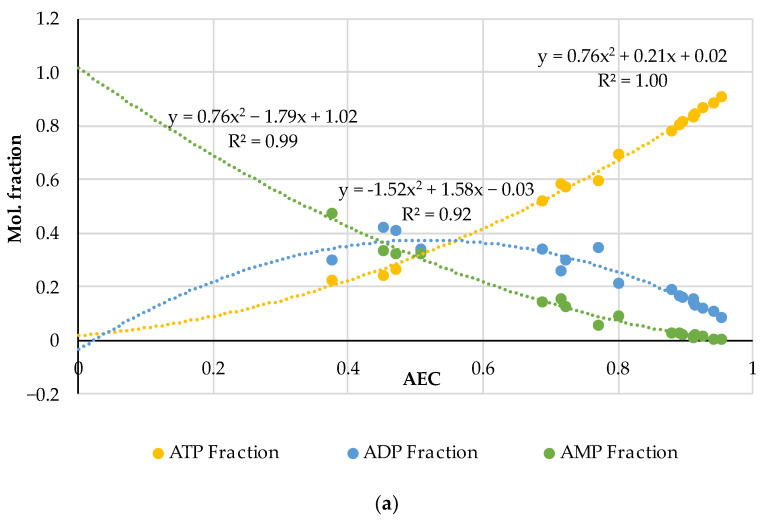

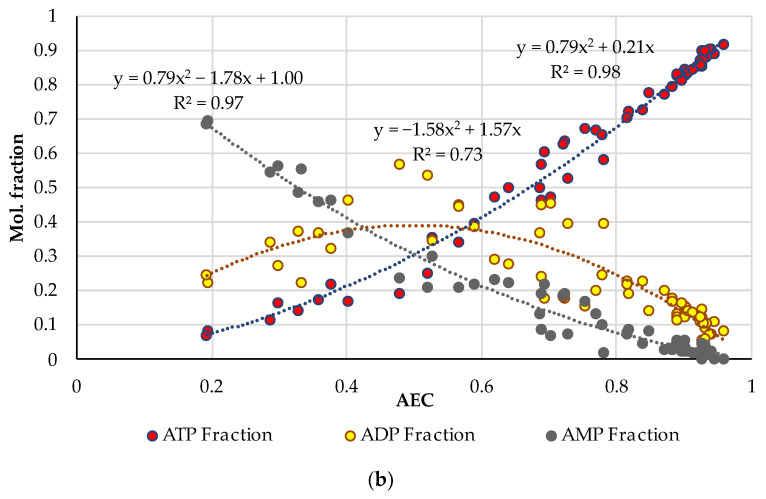
Distribution of adenylates as the mole fractions of ATP, ADP and AMP on the AEC values in 19 samples of very fresh raw pork (**a**) and 61 samples of fish flesh (**b**) with the quadratic polynomial approximations of the experimental curves for ATP (the equation on the right), ADP (in the middle) and AMP (on the left). The diagrams were produced purely by the re-analysis of the experimental data from the original papers [24,35,36,37,38,39,40,41,42,43,44,45,46,47,48].

The AEC index can be applied to the experimental data in another way, namely, to graphically represent the distributions of adenylates in accordance with Formula (4) and compare these with the diagrams in the fundamental works [21,22] that gave rise to the AEC paradigm. For this purpose, the AEC values estimated from the experimental data on adenalyte content in fresh pork and fish presented in a wider set of original works [24,35,36,37,38,39,40,41,42,43,44,45,46,47,48] were plotted along the abscissa axis, and the corresponding values of adenylates relative concentrations expressed in the mole fractions of the total concentration:

(5) ATP Fraction=ATP/(ATP+ADP+AMP)ADP Fraction=ADP/(ATP+ADP+AMP)AMP Fraction=AMP/(ATP+ADP+AMP

were plotted along the ordinate axis. A combined diagram representing the distribution of adenylates on the AEC values for 19 samples of very fresh pork (the initial datasets of ATP, ADP and AMP concentrations are acquired from the original works [35,36,37,38,39]) is shown in Figure 1a. A similar distribution for 61 samples of fresh fish of different species (based on the original works [24,40,41,42,43,44,45,46,47,48]) is presented in Figure 1b. Quadratic polynomial approximations of the experimental distribution curves were added to the diagrams for an easier interpretation. The general patterns are important here, and more detailed information about the pork and fish samples used for this analysis can be found in the corresponding original papers [24,35,36,37,38,39,40,41,42,43,44,45,46,47,48].

Let us emphasize first of all that the classical distributions of adenylates in the basic works [21,22,23] are given for living biological cells, in which these distributions are produced by enzymatic reactions. In our case, in Figure 1, we are dealing only with the extracellular, so-called, free adenylates in fresh but dead biological tissue, directly measured in the experiments with technical equipment. To the best of our knowledge, this is the first time that Atkinson’s theory has been used in such a context. This approach is central to our research considering the influence of thermal processing on adenylate nucleotides.

**Figure 2:**
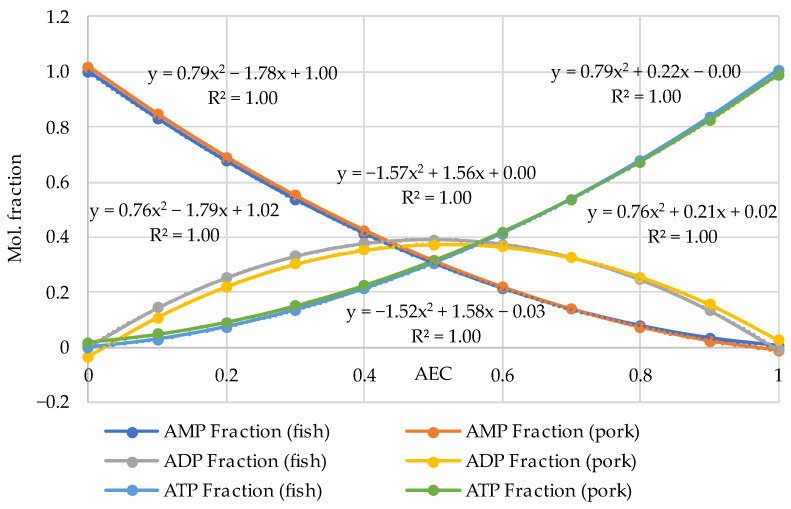
Comparison of the empirical–theoretical approximation of the adenylates distributions for raw pork and fish flesh in the pairs ATP (fish)—ATP (pork), ADP (fish)—ADP (pork) and AMP (fish)—AMP (pork). The equations of the polynomial approximations are given inside the diagram, for fish (above the curves) and pork (below the curves).

The comparison of the distributions for pork and fish made in the pairs ATP (fish)—ATP (pork), ADP (fish)—ADP (pork) and AMP (fish)—AMP (pork) using the analytical functions for every component is given in Figure 2. For this aim, the equations of the polynomial approximations from Figure 1a for meat and Figure 1b for fish were used and the diagrams were plotted with a step of AEC = 0.1. Figure 2 demonstrates that the distributions are almost identical in the fish/pork complementary pairs. Slight differences are observed only for the marginal values AEC ≈ 0 and AEC ≈ 1.0 for ADP. The lower accuracy for ADP (see also Figure 1) is a phenomenon that we perceive here without going into detail. However, the correlation coefficients for all three adenylates exceed 0.9998.

The results presented in Figure 2 reveal a high correlation between theoretical and empirical data and are of great methodological significance, as they indicate the possibility of conducting experiments with mammals’ meat and fish and analyzing the results without making an artificial difference between them. We have already pointed out this possibility previously [32,33]. 

Note here some characteristics of Atkinson’s distribution. It follows from Equation (4) that the AEC values are concentrated in the range from 0 to 1. Theoretically, if AEC = 0, [ATP] = 0 and [ADP] = 0 only [AMP] = 1.0 in the sample. Alternatively, if AEC = 1.0 and [ATP] = 1.0, both [ADP] = 0 and [AMP] = 0 have zero concentrations. Almost exact coincidence of the experimental values in Figure 1 and Figure 2 with the marginal theoretical values indicates the high quality of the experimental data array in [24,25,35,36,37,38,39,40,41,42,43,44,45,46,47,48]. 

The distributions of ATP and AMP have strictly opposite trends and intersect at the point AEC = 0.5. Near this intersection point the three mole fractions are approximately equal to 1/3. The ADP curve has the maximum at AEC = 0.5, which is located slightly above the intersection point of the ATP and AMP curves. The mole fraction values for [ADP] sometimes lie much higher than the 0.4 (40%) point due to large deviations in the experimental data.

An important feature of the distributions of the experimental data is their asymmetry as in Figure 1, namely, a much smaller number of dots for the region AEC < 0.4 than in the area of the higher AEC values. The lack of datapoints at AEC < 0.4 may be caused by the faster disappearance of ATP compared to ADP and AMP and, accordingly, by technical difficulties in the recording of disappearing ATP during the storage of non-frozen meat or fish objects. Even freezing below −60 °C does not stop the ATP degradation (see an example for carp fish in [49]). In [48], there are rich data on how intensively diminishes the ATP concentration during the storage at −7 °C for 24 h in eight fish species. For instance, in Japanese jack mackerel [ATP] dropped by 38.5 and [ADP] 2.5 times, whereas [AMP] increased by 2 times.

### 3.2. The AEC Index of Thermally Processed Pork, Beef, Poultry, and Fish

Various samples of raw and thermally processed food products (in total 34 samples), including pork (2 samples), poultry (6 samples) and fish (26 samples of 5 species, i.e., pollock, salmon, trout, cod, canned sardine), were analyzed by NMR spectroscopy to determine the concentrations of basic nucleotides. Before the NMR technique was applied, all the samples were preliminarily tested for the presence of ATP by the bioluminescence method; the effects of refreshing (nucleotide salvage) were rapidly assessed on spot by the FPMLC method/device [32,33].

**Figure 3:**
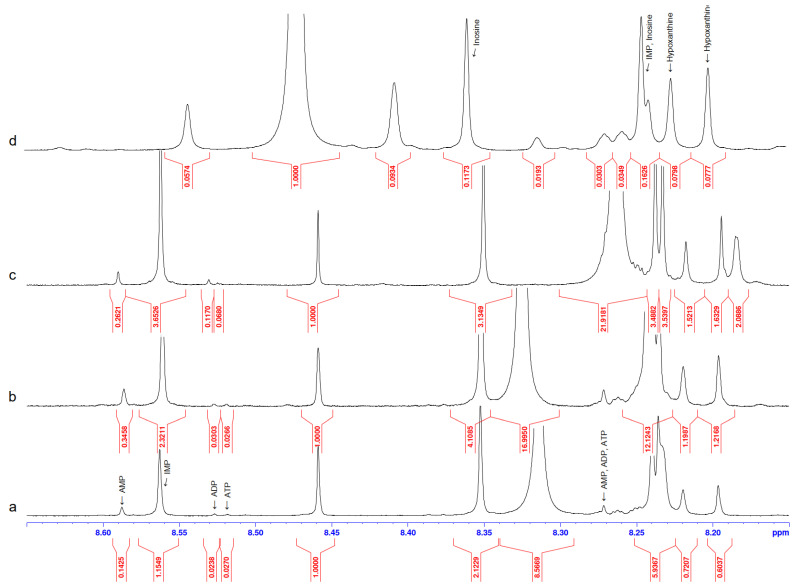
^1^H NMR spectra for 4 meat samples: (**a**) ready-to-eat chicken schnitzel; (**b**)—the same schnitzel after irradiation in microwave oven at 400 W for 1 min; (**c**)—broiler filet boiled in broth at 75 °C for 20 min; (**d**)—broiler filet raw at 24 °C.

Figure 3 shows the NMR spectra of ready-to-eat chicken schnitzel and broiler filet before and after heat treatment. The concentrations of nucleotides and nucleosides in these selected samples are presented in Table 2, which also includes similar data for minced pork (2 samples).

For chicken meat, the most significant increase in the ATP content as a result of heat treatment was observed, e.g., in the ready-to-eat (consumable) chicken schnitzel, the ATP concentration was the highest—0.009 µmol/mL. ATP and ADP are very sensitive to irradiation in a microwave oven, which is almost essential for meat product consumption in our everyday lives. In this study, the concentrations of ATP and ADP in the pork cutlets increased 3- and 4-fold, respectively. At the same time, AMP is a much more stable adenylate and its average concentration for all five experimental cases presented here is characterized by a relatively small deviation Stdev 0.06 ± 0.01 µmol/mL. IMP and other nucleosides are highly resistant to microwave irradiation, especially hypoxanthine; irradiation of the pork cutlets did not change the concentration at all. This circumstance can be viewed in a positive way since hypoxanthine is a substance that gives food a bitter taste [50]. At the same time, AMP enhances the synergistic effect that creates a pleasant umami taste [51]. It is important that the AEC index in Table 2 has low values; the typical value for dead biological tissue is AEC < 0.5.

**Figure 4:**
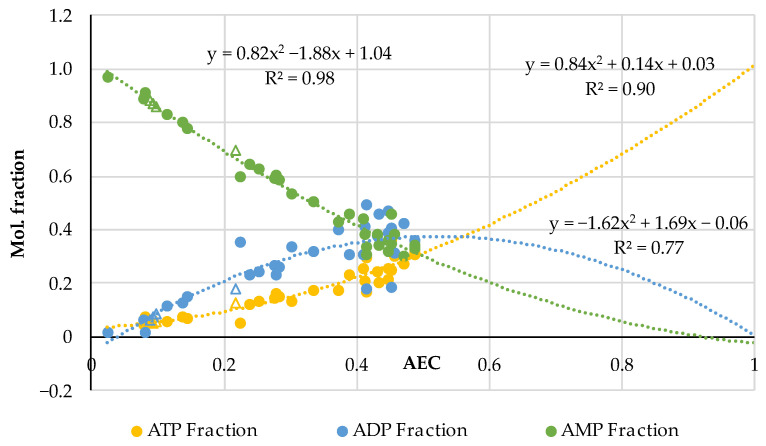
Real distribution of adenylates (dots) and its extrapolation for thermally processed pork, beef, poultry, and fish. In addition to the datapoints obtained in this work by the NMR method (36 triads), 4 triads of datapoints for AEC = 0.218; 0.098; 0.0920; 0875 (triangular markers) for minced boiled beef are adopted from the HPLC results presented in [52].

The results obtained by the NMR measurements for all heated samples (36 triads) are summarized in Figure 4; we supplemented our experimental results with the data (four triads with AEC = 0.218; 0.098; 0.0920; 0.0875) for boiled minced beef obtained by the HPLC method in [52]. We used the same data analysis algorithms as in Section 3.1: the mole fractions of adenalytes were estimated according to Equation (5) and AEC values according to Equation (4). As one can see, the HPLC data on beef coincide perfectly with the distribution for the main group of our NMR data demonstrating the fact of concordance of results obtained by different methods on different kinds of samples.

The effect of asymmetry in the distribution of adenylates relative to the center of the diagram in Figure 4 for thermally processed meat and fish is especially pronounced, but this asymmetry is different from the distribution in Figure 1 plotted for the raw samples. In the “hot” case, we could not register adenylates with the AEC values > 0.5. 

We have carried out a special experiment (see also [53]) with fresh salmon (*Salmo salar*) frozen at −18 °C, which had the AEC = 0.684. After heating in water steam in different modes and different extracts collection the AEC was in the range of 0.488–0.3, i.e., it was always in the “dead zone” with low AEC < 0.5. Evidently, there is a need for more experiments with the heating of absolutely fresh mammal meat and fish to obtain information on the transformation of adenylates in such an exclusive condition. This information could also be useful for medical science considering the regeneration of biological tissue after thermal burns. Such experiments were, however, beyond the scope of this work. 

**Figure 5:**
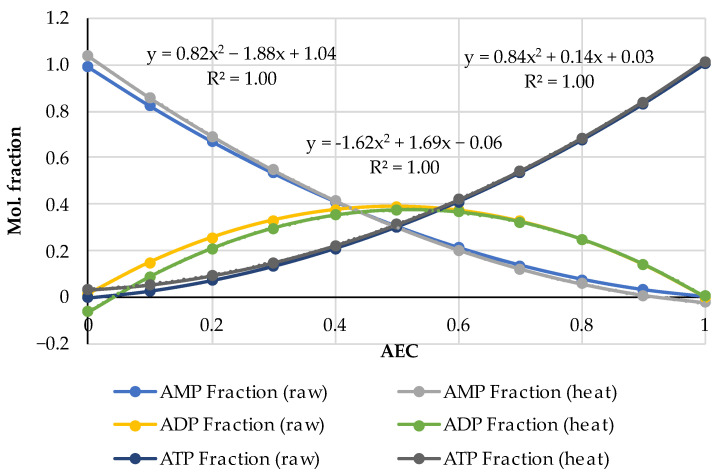
Comparison of the combined empirical–theoretical approximations of the adenylate distributions for raw and heated fish and meat: one triple dataset is based on the combined data for raw fish and meat (Figure 1) produced from literature data (based on 80 samples in total) and the other is for heated fish and meat (Figure 4) produced from the experimental data in the framework of this research (based on 36 samples in total).

In Figure 5, there are drawn the adenylates distributions of two types in parallel: one triple dataset is based on the combined data for the raw fish and meat from Figure 1 and the other is for the heated fish and meat from Figure 4 but with the extrapolation with the step AEC = 0.1 corresponding to the formulas in Figure 4 up to the limit value AEC = 1.

As one can see in Figure 5, the distributions of ATP and AMP for raw and heated meat and fish are in perfect concordance (almost identical) inside each pair. For ADP, there is a minor deviation in the distributions at the lower AEC < 0.5, which resembles the similar effect observable in Figure 2 for raw fish-meat specimens. So, a good complementarity of both distributions, i.e., for the raw samples and for the heated ones, is observed. Such a universal validity in circumstances with avoided enzymatic reactions manifests once again the fundamental rule that the mole fractions of ATP, ADP and AMP are mutually compatible and fixed for any particular value of the AEC.

### 3.3. Nucleotide-Based Freshness Indices of Canned Fish

Various samples of canned fish of different species were bought from local supermarkets; to avoid any interference from added components “au naturel” varieties were chosen when possible. The FPMLC chromatograms of 18 samples were processed according to the protocol described in Section 2.1. The Time index values were in a wide range from 138 to 190 s (Table 3) which leads to the suggestion that the freshness of the raw fish at the moment of manufacturing could vary considerably.

Four of the most representative samples belonging to different categories of freshness according to the Time index were selected for more detailed physicochemical analysis: *Sardina pilchardus* (Mediterranean sardine) from Connétable (Marocco); *Oncorhynchus mykiss* (Rainbow Trout) from Ecofood (Armenia), *Sardinops melanostictus* (Iwashi) and *Oncorhynchus gorbuscha* (Pink Salmon) from Moreslav (Russia).

**Figure 6:**
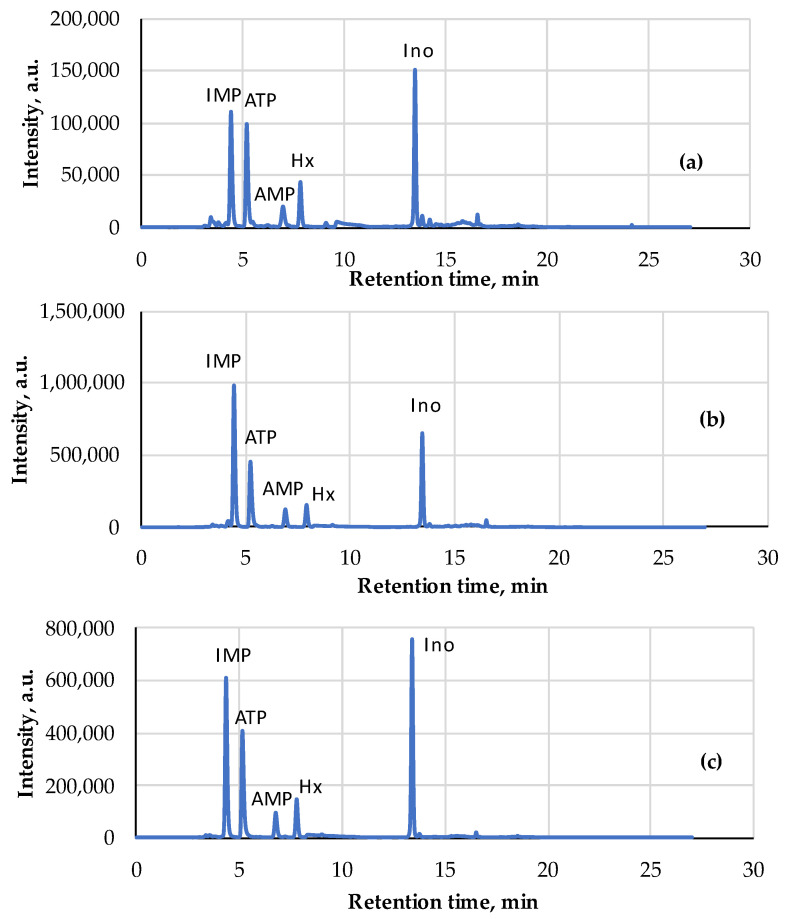

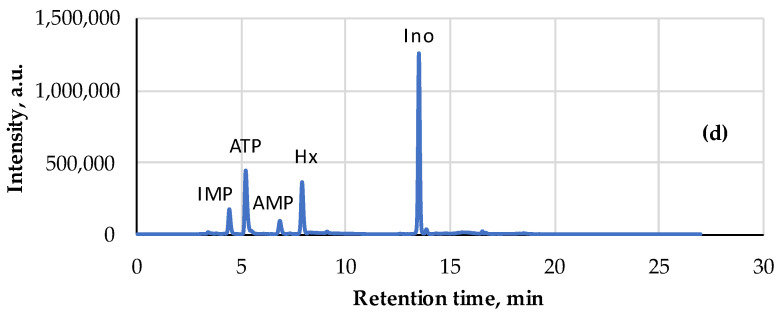
HPLC chromatograms of four canned fish samples: (**a**) Iwashi (544); (**b**) Mediterranean Sardine (545); (**c**) Rainbow Trout (546); (**d**) Pink Salmon (547).

**Figure 7:**
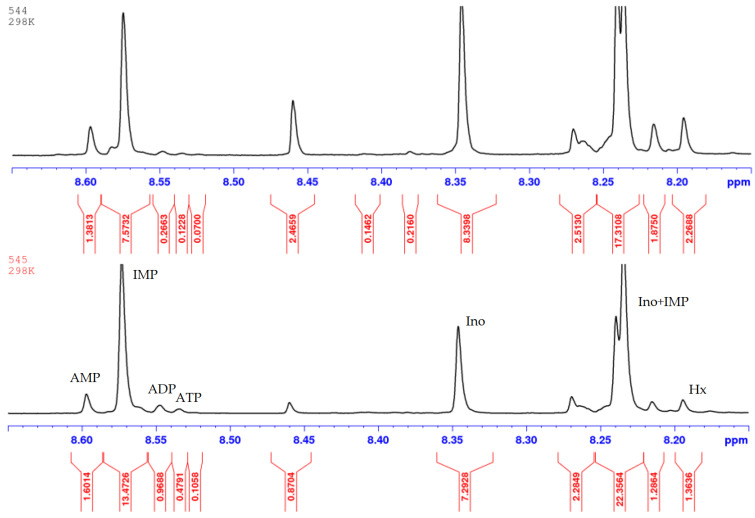
^1^H NMR spectra of two canned fish samples: Iwashi (544); Mediterranean Sardine (545).

**Figure 8:**
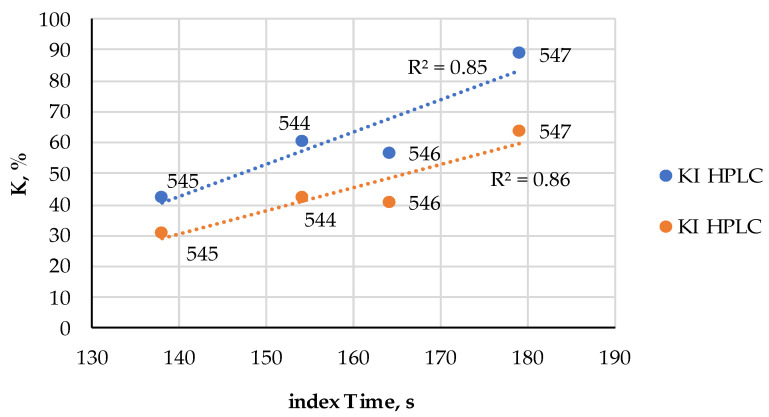
The comparison of the index Time measured by the FPMLC method, and the *K* and *K*_I_ indices measured by the HPLC for four samples of canned fish (the sample codes are indicated next to each point).

**Figure 9:**
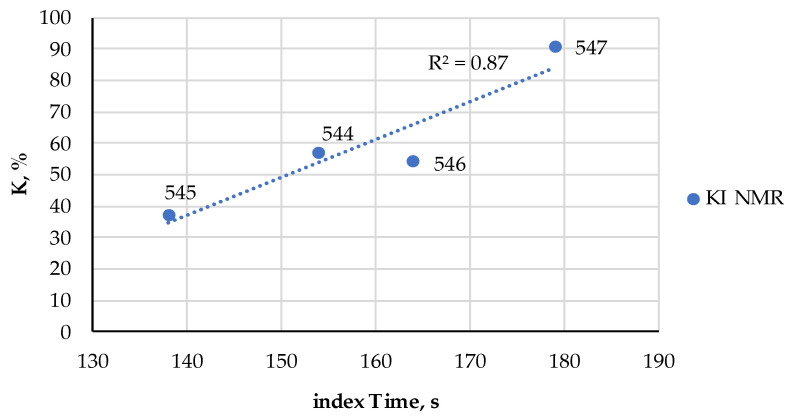
The comparison of the index Time measured by the FPMLC method, and the *K*_I_ index measured by the NMR spectroscopy for four samples of canned fish (the sample codes are indicated next to each point).

The freshness indices AEC, *K* and *K*_I_ of these samples were estimated from the HPLC chromatograms (Table 4, Figure 6) processed according to Section 2.4 and the NMR spectra (Table 4, Figure 7) registered according to Section 2.5 in IBCh RAS. A strong correlation ($R^{2}>0.85$) between the Time index (FPMLC) and *K* and *K*_I_ freshness indices (HPLC, NMR) was observed (Figure 8 and Figure 9).

The high concordance between the FPMLC and HPLC data makes the index Time a good candidate as a viable indicator of the freshness state of raw fish before canning, which could be rapidly assessed by the FPMLC technique using the simple and affordable chemical sensor [33]. It was mentioned earlier that the dietary nucleotides are prone to thermal degradation in the process of high-temperature sterilization treatment before canning and this effect must be taken into account to ensure a correct interpretation of the nucleotide-based freshness indices including the Time index in case of ready-to-eat and canned food. A model experiment with a freshly caught carp fish was conducted to reveal how high-temperature sterilization changes the Time index at different stages of cold storage. 

### 3.4. Influence of High-Temperature Sterilization on Nucleotide-Based Freshness Indices at Different Stages of Cold Storage

A farmed carp fish (*Cyprinus carpio*) was bought from an aquarium at a local supermarket and delivered to the laboratory as quickly as possible. The first measurements were taken within an hour after capture. The fish was put in a refrigerator and stored at a temperature of +2–+4 °C in a closed plastic container for a period of 15 days. The FPMLC chromatograms were processed for the samples of the raw fish and the samples, which were steam-cooked in a pressure cooker at 114–120 °C, on days 1–6, 8–11 and 15 to imitate the high-temperature sterilization treatment before canning. The samples were taken from the carcass and stored in the refrigerator immediately before the preparation of liquid extracts. At least two samples were taken from different parts of the carcass for each measurement (in total, 22 raw and 22 thermally processed samples were analyzed); the results were averaged. Additionally, on day 2, a bigger piece of muscle tissue was thermally treated and stored in the refrigerator until the end of the experiment. The FPMLC chromatograms of the samples taken from this piece (in total, 22 samples) were processed on days 3–6, 8–11 and 15.

**Figure 10:**
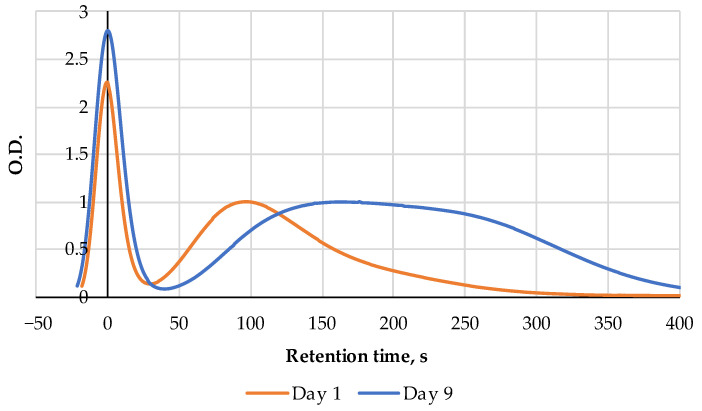
Comparison of two FPMLC chromatograms processed on days 1 and 9 of the cold storage for the samples of the raw carp fish.

The FPMLC chromatograms of the raw fish samples show a gradual increase in the Time index in the course of the cold storage. On days 8 and 9 of the experiment, a bifurcation of the peak associated with the ATP metabolites became noticeable, which could be associated with the increasing hypoxanthine concentration at the early stages of bacterial spoilage (Figure 10). Starting from day 10, the bifurcated peak gradually shifted to the right. On the 15th day of measurements, when the fish was completely spoiled, the index Time reached a value of 261 s, which is close to the index Time for pure hypoxanthine. 

**Figure 11:**
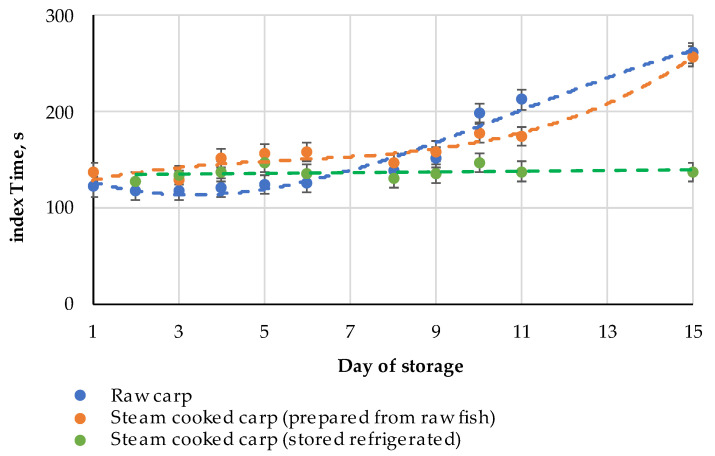
Dynamics of the index Time for the raw carp fish in the process of the prolonged cold storage and steam-cooked carp fish (immediately prepared from the cold stored raw fish and stored refrigerated after heating of the fresh fish at the beginning of the experiment); the experimental datasets are approximated with polynomial functions for an easier interpretation.

The dynamics of the index Time for the raw and steam-cooked fish are presented in Figure 11. When muscle tissue was steam-cooked on the first days of the experiment, higher values for the index Time were observed compared to the raw fish due to the nucleotide thermal degradation. On the 10th day of measurements and further on, the Time index for the thermally processed fish became lower compared to the Time index values for the raw fish, apparently due to the effect of nucleotide salvage. On the 11th day, the second peak on the chromatogram demonstrated bifurcation after steam cooking, while no peak bifurcation was observed on the chromatogram of the raw fish. A similar situation was observed on the 15th day, but the right-side peak became more intense than the left-side one.

The sample, which was steamed on the second day of the experiment and cold stored afterward, showed fluctuations of the Time index values in the range of 131–147 s (Figure 11). As expected, there was no increase in the Time index values during the storage, because of the enzyme deactivation. It once more proves the fact that the nucleotide-based freshness indices determined for ready-to-eat and canned fish products indicate the freshness status of the raw material before manufacturing rather than the product itself. Dependence of the index Time for the steam-cooked carp on the storage period of the raw fish before the heat treatment (Figure 11) was used to roughly estimate the storage time before canning for the four samples from Table 4. It is necessary to emphasize that such dependence may vary for different fish species and using the experimental data obtained for carp for the canned fish samples of different species is not completely justified, so the results presented in Table 4 should be considered only as a demonstration of the feasibility of the suggested approach. 

**Figure 12:**
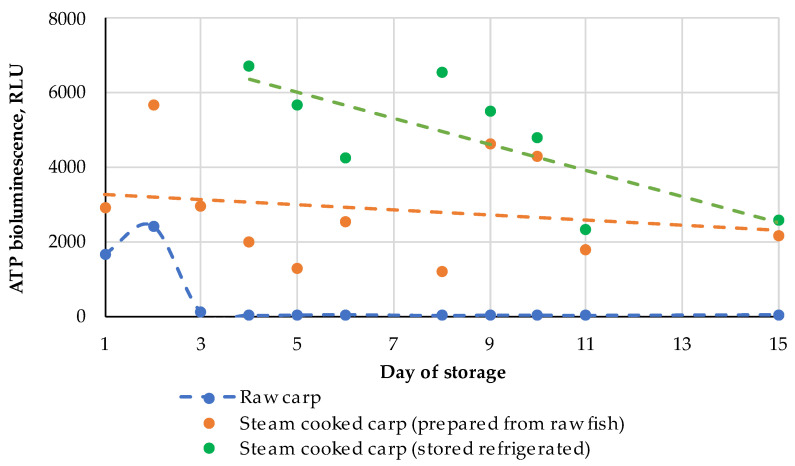
Dynamics of the ATP concentration determined by the bioluminescence assay for the raw carp fish in the process of prolonged cold storage and steam-cooked carp fish (immediately prepared from the cold stored raw fish and stored refrigerated after heating of the fresh fish in the beginning of the experiment); the experimental datasets are approximated with linear functions for an easier interpretation.

The dynamics of the ATP content in raw and steamed fish samples were measured by the bioluminescence technique presented in Figure 12. Despite high fluctuations, which are typical for this technique, the general trends are quite evident. ATP in the raw fish decreased to a near-zero level after 45 h of storage; quite unexpectedly, a high level of free ATP was observed even after 24 h after catching. After heat treatment the free ATP content was higher than in the raw samples even in the first days of storage; moreover, it decreased very slowly up to the 15th day of the experiment. In the cold-stored sample prepared on day 2, the content of ATP decreased very slowly compared to the raw samples.

## 4. Discussion

The concentrations of free adenylates restored by heating in different types of meat (presented in Table 2) take values in the range of 0.001–0.01 µmol/mL, which are slightly lower than the values previously observed in pollock, shrimp, beef, and pork (see Table 3 in our work [32]). However, it is safe to say that there are no fundamental quantitative differences between the results presented here and our earlier experiments considering the different types of food products and different methods of thermal treatment. Moreover, based on the datasets obtained here and in the previous work [32], we can conclude that in mammal, poultry, fish, and marine invertebrate meat, the restoration of free adenylates and their subsequent presence in significant concentrations within the µmol/mL order of magnitude is apparently quite typical for ready-to-eat food.

**Figure 13:**
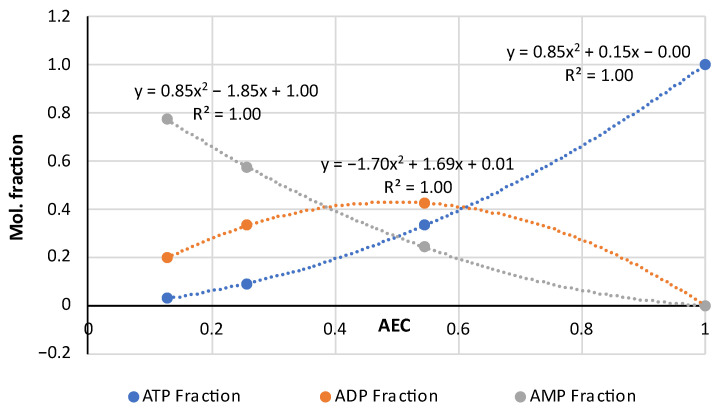
Thermal degradation of ATP at +80 °C and relative distribution of ADP and AMP as the products of this reaction. The dots from the right to the left correspond to the values measured at the very beginning of the experiment (AEC = 1.0) and at 8, 16 and 24 (AEC = 0.129) hours later on.

At this stage, one can say that we have to deal with a two-fold effect: firstly, the reappearance of vanished adenylates in fish and meat during their ordinary heating after storage. Secondly, the stable presence of adenylates is not in a chaotic conglomerate but in the ordered triads ATP + ADP + AMP distribution according to the adenylate energy charge rule. The situation is interesting also in an additional dimension: such typical and important metabolites as adenylates can reappear and persist for quite a long time in the enzyme-free environment. Therefore, a question arises: how can be the AEC approach substantiated by the other experiments with adenylates in an enzyme-free environment? Fortunately, there is information on the experiments performed with heating of ATP, ADP and AMP in the absence of enzymes [54,55]. ATP solutions in media with different pH values were heated and ADP and AMP, the products of the thermal degradation of ATP, were monitored for 24 hours [55]. We have used the results obtained for pH = 4 in [55] and represented them in the spirit of the AEC approach in Figure 13.

**Figure 14:**
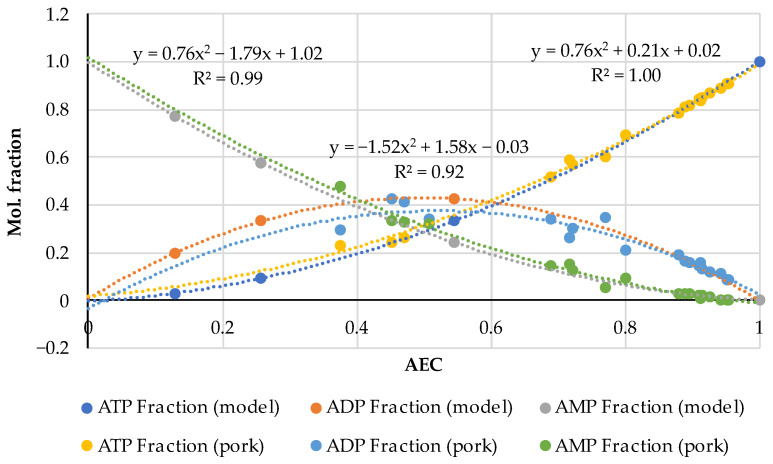
Combination of the molar distributions of adenylates in the enzyme-free model solution and in the intercellular fluid of absolutely fresh pork according to the data in Figure 1a. The experimental points are given together with the extrapolation curves; the approximation equations are given for the pork datasets.

The relative molar distributions of ATP, ADP and AMP in the heated enzyme-free model solution [55] and in the intercellular fluid of absolutely fresh pork [37] are presented in Figure 14 for a comparison. As one can see, the concordance of both distributions for the pH = 4 is extended almost over the whole range of the AEC values. The distributions for ATP and AMP are almost identical whereas the concordance for ADP is not so good because of the absence of data for ADP in pork with the AEC < 0.3. Nevertheless, there is not much doubt about the compatibility of these two distributions. 

Such an overlapping in the distributions of adenylates in fundamentally different liquid media demonstrates the inherent ability of adenylate molecules to mutually coordinate each other and create a ternary composition in accordance with the AEC value. This phenomenon deserves special attention, which, however, goes beyond the scope of this study.

Apart from a purely theoretical interest, a deeper understanding of the thermal transformations of the ATP-related compounds in different types of meat and fish is extremely important for the evaluation of the freshness state of ready-to-eat and canned food products, which are increasingly popular in our fast-moving world. It is a commonly accepted problem in many countries that meat and fish on the edge of spoilage are sometimes used as raw material for the manufacturing of ready-to-eat and canned food, while an unpleasant flavor and taste are masked with strong spices. Conventional nucleotide-based freshness indices including the *K, K*_I_, *H* and the simplified Time index introduced in our earlier works in conjunction with the FPMLC technique are ideal for identifying such unfair tricks due to the fact that after thermal processing the relative content of the dietary nucleotides and nucleosides changes very slowly if changes at all considering enzyme deactivation. As a result, the values of these indices indicate the freshness category of raw meat or fish before processing rather than the storage time of the final products (dishes). 

It was shown that the index Time extracted from the FPMLS chromatograms of different canned fishes strongly correlates with the *K* and *K*_I_ indices and could be used as a simpler and cheaper alternative to the HPLC and NMR techniques. For the correct interpretation of these indices as applied to ready-to-eat or canned food, two crucial effects occurring during the culinary processing of dishes or autoclaving before canning must be taken into account: the thermal degradation of nucleotides (an increase in the values of the indices) and nucleotide salvage (a decrease in the values of the indices). The latter effect is more prominent for low-quality meat and fish on the edge of spoilage.

Thus, the ATP metabolites are essential nutrients that are important both for a pleasant taste and high nutritional value of fish and meat food. Their relative content is a good indicator of the freshness category of not only raw products but also of ready-to-eat and canned food. Quite surprisingly, ATP and the related compounds were detected in food products even after high-temperature processing; moreover, it was revealed that adenylate molecules can mutually coordinate in accordance with the AEC value.

## 5. Conclusions

This paper is a continuation of the earlier works [32,33] aimed at investigating the thermal effects in mammal, poultry and fish meat during conventional cooking processes. The emphasis in these studies is put on the nutritional nucleotides and nucleosides being, as substantiated in the present research, not an occasional part of traditional meals. A variety of analytical methods for nucleotides and nucleosides detection and quantitate determination was used, which included both sophisticated laboratory techniques such as NMR and HPLC and rather simplified ones that could be used even outside the laboratory environment. The latter include the well-known ATP bioluminescence assay as well as a reliable and inexpensive FPMLC sensor designed for the first time by the authors [33]. 

Nowadays, ready-to-eat and canned food products manufactured on an industrial level have reached a significant proportion in the total consumption of meat and fish in developed countries and, apparently, it constantly grows. Considering this trend, it is very important to develop rapid and affordable methods for testing the freshness and nutritional value of such products. While the freshness indices based on the ATP metabolites are widely used for the quality assessment of raw meat and fish, it is not completely clear to what extent they can be applied to thermally processed ready-to-eat and canned food. It is even more unclear whether it is possible to quantify the consumer value of foods based on the content of free adenylates in them, but it is certain that ignoring the presence of adenylates in them is not correct. This is a kind of a challenge since ready-to-eat food is rather *terra incognita* in the sense of knowledge on the transformation of the nutritional adenylates during the hot preparation of meat and fish dishes. To date, in this regard, the available data are scattered and do not form a body of knowledge. In this pilot work, we observed that animal, chicken and fish meat may contain ATP and other adenylates at the level of 0.001–0.01 µmol/g, but at the same time, in a number of products, we were unable to detect even traces of ATP (we do not disclose these data in details as preliminary ones). The ATP metabolites which are not adenylates are always present in ready-to-eat and canned food, but their concentrations indicate a rather fresh state of the raw materials just before preparation than the freshness of the dishes themselves: the fact that was discovered before in some specific cases [27] and confirmed in this work. These subjects and the effects occurring in them should be investigated more systematically. 

In the initial state, there is still virtually no information about the eventual significance of the adenylate energy charge (AEC) when assessing the quality of food products prepared from fish and meat of domestic animals. Nevertheless, it seems that the AEC ideology could be tried in respect to *Haute cuisine* and nutritionists should take an interest in the situation and develop an assessment system in which there would be a place for the AEC index.

Moreover, animal meat and fish meals are very often prepared in combination with plant components and extracts from them, for which there are almost no data on the effect of heating on the existence of adenylates and on their properties in them. This problem could be a subject for future research important not only for vegetarians but for all people.

## Figures and Tables

**Table 1:** The AEC values for the various categories of absolutely fresh pork according to data in [35,36,37,38,39].

Pork	AEC	Storage, Hours	Ref.
Normal (averaged)	0.85	1–2	[35,36,37]
Low quality	0.715	2	[37]
PSE	0.65	1	[35]
DFD	0.6	1	[35]
Normal	0.91	2	[38]
Heterozygote	0.77	2	[38]
Wild-type	0.90	0	[39]
AMPK	0.93	0	[39]

**Table 2:** Content of ATP, ADP, AMP, IMP, Ino, Hx and AEC in the meat samples of different condition (in µmol/mL).

Meat	ATP	ADP	AMP	IMP	Ino	Hx	AEC	Condition
Broiler file	0.005	0.036	0.061	0.67	0.65	0.32	0.225	Boiled 75 °C 20 min
Chicken schnitzel	0.009	0.017	0.047	0.41	0.77	0.2	0.24	Bought as ready-to-eat food
Chicken schnitzel	0.007	0.012	0.075	0.45	1.0	0.24	0.138	After MW at 400 W 1 min
Minced pork balls	0.001	0.001	0.06	0.22	0.36	0.24	0.024	Bought as ready-to-eat food
Minced pork balls MW irradiated	0.003	0.004	0.057	0.21	0.39	0.24	0.078	After MW at 400 W 1 min

**Table 3:** Time index values for the samples of canned fish.

Sample #	Fish Specie	Time, s	HPLC/NMR Sample Code
1	Tuna	176	
2	Tuna	188	
3	Pink Salmon	172.5	
4	Pink Salmon	187.5	
5	Mediterranean Sardine	138	545
6	Rainbow Trout	189.5	
7	Sockeye	165	
8	Mackerel	164	
9	Salmon	192.5	
10	Chum Salmon	188.5	
11	Rainbow Trout	180	
12	Pink Salmon	168.5	
13	Tuna	164.5	
14	Atlantic salmon	171	
15	Coho Salmon	156.5	
16	Trout	164	546
17	Iwashi	154	544
18	Pink Salmon	178.5	547

**Table 4:** The nucleotide-based freshness indices and estimated storage period of raw materials used for the manufacturing of canned fish.

Canned Fish	Index Time, s FPMLC	*K*_I_, HPLC	*K*_I_, NMR	*K*, HPLC	*K*, NMR	AEC, NMR	Estimate Storage Period before Canning at +2…+4 °C, Days
Iwashi (Moreslav)	154	61	57	42	53	0.11	3–7
Mediterranean sardine (Connétable)	138	43	37	31	34	0.28	0–2
Rainbow trout (Ecofood)	164	57	54	41			3–7
Pink salmon (Moreslav)	179	89	91	64			>8

## Data Availability

The data presented in this study are available on request from the corresponding author due to Ldiamon AS commercial secrets.
